# Supplementary material for: Complexome profiling of the Chlamydomonas psb28 mutant reveals TEF5 as an early PSII assembly factor
Source: Plant Cell. 2025 Mar 17;37(6):koaf055. doi: 10.1093/plcell/koaf055 (PMC12164590; doi:10.1093/plcell/koaf055)
Supplement: koaf055_Supplementary_Data [file koaf055_supplementary_data.zip › psb28_interactive.zip!/plots/intensity/qualityPlots/peptides/CGL52_Cre12.g536800.t1.2.html]

### Cre12.g536800.t1.2

Trivial name: CGL52  
SeqCoverage [median]: 23.60  
Mass: 30.24 kDa  
  
 

| Sequence | MissedCleavage | Mass [Da] | wtIntensity | muIntensity | Score |
| --- | --- | --- | --- | --- | --- |
| GRYAAATLYQPGVTYSDGFR | 1 | 2192.07 | 1413.10 | 2597.86 | 98.17 |
| LGGGGGADMNNLPSDPTR | 0 | 1727.79 | 7877.50 | 24539.03 | 176.89 |
| QAVEDAKEGLTK | 1 | 1287.67 | 7158.79 | 10428.38 | 93.77 |
| TFTGVEGYK | 0 | 1000.49 | 0.00 | 492.05 | 54.16 |
| TFTGVEGYKR | 1 | 1156.59 | 9262.76 | 30067.22 | 97.46 |
| YAAATLYQPGVTYSDGFR | 0 | 1978.94 | 1233.77 | 23199.94 | 188.29 |
| YAWSAK | 0 | 724.35 | 0.00 | 1367.09 | 80.56 |
|  |  |  | 26945.9 | 92691.6 |  |
